# Intervention of cGAS‒STING signaling in sterile inflammatory diseases

**DOI:** 10.1093/jmcb/mjac005

**Published:** 2022-01-27

**Authors:** Ze Hong, Jiahao Mei, Hanli Guo, Juanjuan Zhu, Chen Wang

**Affiliations:** State Key Laboratory of Natural Medicines, Department of Life Science and Technology, China Pharmaceutical University, Nanjing 211198, China; State Key Laboratory of Natural Medicines, Department of Life Science and Technology, China Pharmaceutical University, Nanjing 211198, China; State Key Laboratory of Natural Medicines, Department of Life Science and Technology, China Pharmaceutical University, Nanjing 211198, China; State Key Laboratory of Natural Medicines, Department of Life Science and Technology, China Pharmaceutical University, Nanjing 211198, China; State Key Laboratory of Natural Medicines, Department of Life Science and Technology, China Pharmaceutical University, Nanjing 211198, China

**Keywords:** cGAS, STING, DNA, sterile inflammatory disease, antagonist

## Abstract

Sterile inflammation characterized by unresolved chronic inflammation is well established to promote the progression of multiple autoimmune diseases, metabolic disorders, neurodegenerative diseases, and cardiovascular diseases, collectively termed ‘sterile inflammatory diseases’. By recognizing host-derived DNA, cyclic guanosine monophosphate–adenosine monophosphate synthase (cGAS) activates endoplasmic reticulum-associated stimulator of interferon genes (STING), which leads to the induction of type I interferons and inflammatory cytokines or immunogenic cell death that promotes sterile inflammation. Additionally, the DNA/cGAS-independent mode of STING activation has also been characterized in the progression of several sterile inflammatory diseases. This review focuses on the molecular mechanism of cGAS-dependent and cGAS-independent STING signaling under various disease conditions, particularly highlighting the diverse initiators upon this signaling pathway. We also summarize recent advances in the discovery of antagonists targeting cGAS and STING and the evaluation of their efficiencies in preclinical models. Finally, we discuss potential differences in the clinical applications of the specific antagonists, which may shed light on the precision therapeutic interventions.

## Introduction

Host pattern recognition receptors recognize conserved pathogen-associated molecular patterns to initiate intricate signaling cascades that activate innate and adaptive immune responses to eradicate microbial infection (Janeway, 1989). As a major cytosolic DNA sensor, cyclic guanosine monophosphate (GMP)‒adenosine monophosphate (AMP) (cGAMP) synthase (cGAS) recognizes viral or bacterial double-strand DNA (dsDNA) in the cytosol that induces a global conformational change for the binding of adenosine triphosphate (ATP) and guanosine triphosphate (GTP) substrates and catalyzes the production of a noncanonical cyclic dinucleotide (CDN), c[G(2′,5′)pA(3′,5′)p] (2′3′-cGAMP) (Ablasser et al., 2013; Civril et al., 2013; Gao et al., 2013a; Li et al., 2013; Sun et al., 2013). As a second messenger, 2′3′-cGAMP binds to endoplasmic reticulum (ER)-associated stimulator of interferon genes (STING, also known as MITA, ERIS, or MPYS) and then causes its higher order multimerization and activation (Ishikawa and Barber, 2008; Jin et al., 2008; Zhong et al., 2008; Sun et al., 2009; Gao et al., 2013b; Zhang et al., 2013; Shang et al., 2019). Activated STING traffics from the ER to the Golgi apparatus and recruits TANK-binding kinase 1 (TBK1) and IκB kinase ε (IKKε), which phosphorylates STING and subsequently interferon regulatory factor 3 (IRF3) and nuclear factor-κB (NF-κB) to induce the transcription of antimicrobial cytokines, including type I interferons (IFNs), inflammatory cytokines, and interferon-stimulated genes (ISGs) (Tanaka and Chen, 2012; Abe and Barber, 2014; Luo et al., 2016; Zhang et al., 2019; Balka et al., 2020). STING activation also induces a noncanonical autophagy process in an IRF3/IFN-independent manner that mediates antiviral responses (Gui et al., 2019; Wu et al., 2020; Yamashiro et al., 2020).

Different from the classical pattern recognition dogma with pathogen-specific patterns, cGAS is incompetent at discriminating self-DNA from non-self-DNA (Ablasser and Chen, 2019; Zierhut and Funabiki, 2020). The recognition of dsDNA by cGAS is in a sequence-independent manner with a preference for long dsDNA (Luecke et al., 2017; Zhou et al., 2018). Extracellular DNA, mitochondrial DNA (mtDNA), and nuclear DNA are capable of activating cGAS when they gain access to it (Ablasser and Chen, 2019). Moreover, cGAS has a wide cellular distribution that involves localization at the inner leaflet of the plasma membrane, cytoplasm, and nucleus (Sun et al., 2013; Barnett et al., 2019; Jiang et al., 2019). Multifaceted safeguard mechanisms have been adopted by the host to prevent aberrant cGAS‒STING signaling-induced autoreactivity under steady-state conditions, which include (i) segregation of cGAS away from extracellular DNA and mtDNA via compartmentalization of cGAS to the cytosol and the inner plasma membrane, (ii) tight tethering of nuclear cGAS by histones to prevent cGAS activation by genomic DNA (Boyer et al., 2020; Kujirai et al., 2020; Michalski et al., 2020; Pathare et al., 2020; Zhao et al., 2020a), (iii) nucleases and phosphodiesterases that degrade immunogenic DNA and 2′3′-cGAMP (Li et al., 2014; Motwani et al., 2019b), and (iv) sophisticated protein‒protein interaction and posttranslational modification (PTM) networks to maintain the stability and functionality of cGAS and STING (Hertzog and Rehwinkel, 2020; Hong et al., 2021a). Disrupting one piece of these regulatory mechanisms leads to the imbalance of cGAS‒STING signaling that induces or aggregates multiple sterile inflammatory diseases, including autoimmune diseases, neurodegenerative diseases, metabolic disorders, and cancers (Bai and Liu, 2019; Motwani et al., 2019b; Paul et al., 2020). Accordingly, pharmacological modulation of cGAS and STING shows a promising therapeutic effect in the preclinical model of such diseases.

Given the critical role of cGAS‒STING signaling in the development of various diseases, drug discovery targeting cGAS and STING has gained much attention from the scientific community and pharmaceutical companies in the past few years (Sheridan, 2019; Decout et al., 2021). Here, we summarize recent progress in the understanding of aberrant cGAS‒STING signaling in sterile inflammatory diseases and in the discovery of novel antagonists targeting cGAS and STING. We highlight diverse initiators upon cGAS and/or STING presenting in these pathological conditions and discuss potential differences in the clinical applications of specific antagonists, which may shed light on precision medicine against cGAS/STING-driven sterile inflammatory diseases.

## DNA‒cGAS‒STING signaling in sterile inflammatory diseases

**Figure 1:**
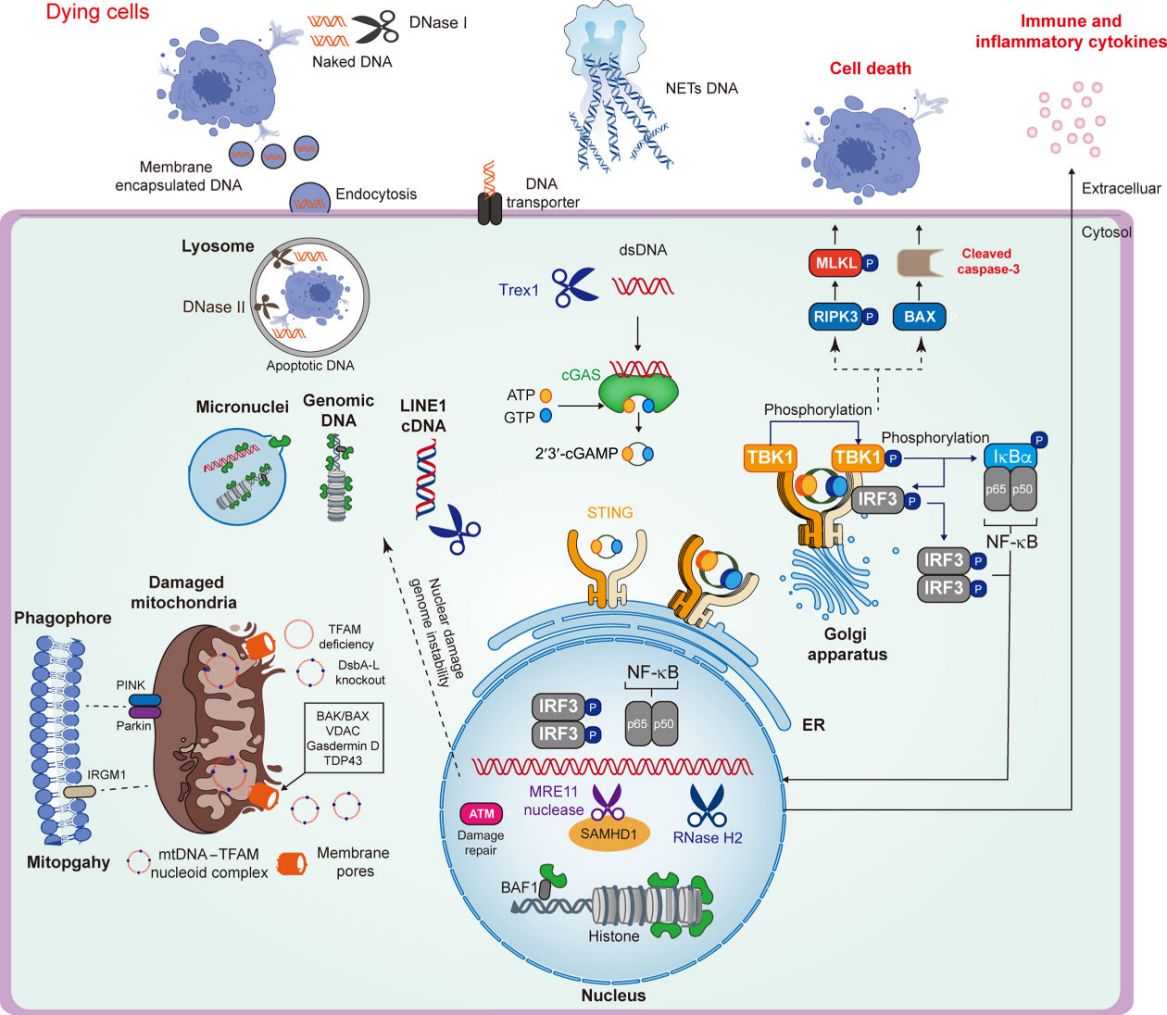
DNA‒cGAS‒STING signaling in sterile inflammatory diseases. Aberrant exposure of extracellular DNA (naked DNA, membrane-coated DNA, or NET DNA), mtDNA, and nuclear DNA (micronuclei, genomic DNA, or LINE1 cDNA) to cGAS under the specifically pathological conditions leads to cGAS activation that catalyzes the production of 2′3′-cGAMP, which further induces the oligomerization and translocation of STING upon binding. Activated STING not only recruits TBK1 and IKKε to induce the transcription of immune and inflammatory cytokines, but also triggers cell death through inducing proapoptotic gene expression. Eventually, these cGAS/STING-dependent cellular responses initiate or aggravate multiple sterile inflammatory diseases.

Disrupting DNA compartmentalization and/or its metabolism leads to cGAS activation in sterile inflammatory diseases (Motwani et al., 2019b). Specifically, under pathological conditions, (i) compromised membrane integrity following mitochondrial stress and chromosomal damage induces the efflux of mtDNA and nuclear DNA (micronuclei) into cytosol (Zierhut and Funabiki, 2020); (ii) extracellular DNA, released from dying cells, overwhelms the cell capability of degradation after being endocytosed and thus gains access to cytosolic cGAS (Lou and Pickering, 2018); and (iii) loss-of-function (LOF) gene mutations in the nucleases (*DNase I, DNase II, Trex1*, and *RNase H2*) with impaired function cause DNA aggregation and cGAS activation (Uggenti et al., 2019). Here, we describe the effect of cGAS-dependent DNA sensing on the progression of sterile inflammatory diseases, focusing particularly on different sources of DNA-driven cellular responses (Figure 1).

### Extracellular DNA

Extracellular self-DNA originates from nucleated cell dying by apoptosis, necrosis, or NETosis. Released as naked DNA, membrane-coated microparticles, or apoptotic bodies from dead cells, extracellular DNA is digested by deoxyribonuclease 1 (DNase1) and its homolog DNase1L3 in serum or degraded by DNase2 in the phagolysosomal compartment when engulfed by macrophages (Lou and Pickering, 2018).

Recently, a growing number of studies have suggested that inadequately digested extracellular DNA in the disease contexts induces activation of the cGAS‒STING pathway. For example, extracellular DNA, released from ischemic cardiomyocyte death during myocardial infarction, exacerbates ventricular dilation, contractile dysfunction, and ventricular rupture by activating cGAS‒STING‒IRF3 signaling in macrophages after being phagocytosed (King et al., 2017). Likewise, DNA from damaged aortic smooth muscle cells or dying acinar cells during sporadic aortic aneurysm and dissection or acute pancreatitis activates cGAS‒STING signaling in macrophages, which promotes aortic degeneration or pancreatic inflammation (Zhao et al., 2018; Luo et al., 2020). These effects are confirmed by adding DNase1 in the dying cell‒macrophage coculture system, which prefers to digest extracellular naked DNA and prevents STING activation in macrophages. DNA in apoptotic bodies is abundant in systemic lupus erythematosus (SLE) serum that facilitates ISG expression via the cGAS–STING pathway (Kato et al., 2018). Consistently, defecting in this DNA degradation by genetic ablation of *DNase2* in mice is embryonic death by overactivation of STING signaling (Ahn et al., 2012). Additionally, NETosis, the death of neutrophils to release neutrophil extracellular traps (NETs), was originally characterized in infectious diseases induced by pathogens but has recently been described in sterile inflammatory diseases induced by cytokines, cholesterol crystals, and autoantibodies (Jorch and Kubes, 2017). Oxidized mtDNA released by NETosis in human lupus promotes autoimmunity and type I IFN signatures via activating the cGAS‒STING pathway (Lood et al., 2016). NET-induced cGAS‒STING signaling also contributes to blood‒brain barrier disruption in ischemic stroke after thrombolysis with tissue plasminogen activator therapy (Wang et al., 2021). In contrast, accumulation of DNA in apoptotic microparticles in *DNase1L3*^–/–^ mice develops SLE syndrome independently of STING activation (Sisirak et al., 2016). These studies raise questions such as what dictates these distinct outcomes and how these extracellular DNAs gain access to cGAS. Answering these questions will need to (i) address the characteristics of extracellular DNA, such as the status of methylation, histone modification, protein interaction, and membrane encapsulation, (ii) explore the effect of these characteristics on the uptake patterns by phagocytes, and (iii) analyze intrinsic relationships between the uptake patterns and DNA recognition by intracellular DNA receptors, such as toll-like receptor 9, absent in melanoma-2, and cGAS, in future studies.

### mtDNA

mtDNA is a type of small, double-stranded circular molecule encased in the outer and inner mitochondrial membranes with thousands of copies presenting in each cell (West and Shadel, 2017). Different from nuclear DNA, mtDNA is packaged into nucleoids by mitochondrial transcription factor A (TFAM) instead of histones and prone to oxidative damage due to the oxidative environment in the mitochondrial matrix (West and Shadel, 2017). Aberrant mtDNA packing due to TFAM deficiency promotes the escape of mtDNA into the cytosol, which induces a type I IFN response by activation of the cGAS‒STING pathway (West et al., 2015). Notably, specific deletion of *Tfam* in renal tubule cells develops metabolic dysfunction and severe renal disease in mice, which models the decreased TFAM expression observed in the kidney of patients with chronic kidney disease (Chung et al., 2019). Mechanistically, kidney inflammation and fibrosis are induced by NF-κB rather than IRF3 activation downstream of the mtDNA‒cGAS‒STING pathway (Chung et al., 2019). Mitochondrial membrane pore formation also allows the liberation of mtDNA into the cytosol during cell death. Apoptotic mtDNA efflux through BAX/BAK-mediated mitochondrial outer membrane pores does not result in cGAS activation due to the cleavage of cGAS by concomitant activation of apoptotic caspases (McArthur et al., 2018; Riley et al., 2018; Ning et al., 2019). Interestingly, it seems that the cleavage of cGAS by inflammatory caspase in pyroptosis is negligible in diseases as is shown in vascular endothelium and retinal pigmented epithelium death, where activated gasdermin D and/or Alu-retroelement RNA induce the formation of mitochondrial pores and release of mtDNA, thus activating the cGAS‒STING pathway and suppressing cellular regeneration (Wang et al., 2017; Kerur et al., 2018; Huang et al., 2020). Additionally, the leakage of mtDNA through the mitochondrial permeability transition pore induced by familial amyotrophic lateral sclerosis (ALS)-associated TDP-43 mutant (Q331K) and voltage-dependent anion channel oligomers activates cGAS‒STING signaling, which drives neuroinflammation and lupus-like disease, respectively (Kim et al., 2019; Yu et al., 2020). Finally, mtDNA release can also be triggered by mitochondrial stress or damage. For example, mitochondrial damage caused by lipid overload in endothelial or Kupffer cells activates the cGAS‒STING pathway and increases tissue inflammation, which is critically involved in metabolic diseases (Mao et al., 2017; Yu et al., 2018). Increased mitochondrial stress in adipose tissues induced by high-fat diet feeding or fat-specific disulfide-bond A oxidoreductase-like protein (DsbA-L) knockout enhances mtDNA release into the cytosol, which leads to adipose inflammation, insulin resistance, and reduced thermogenesis by activation of the cGAS‒STING pathway (Bai et al., 2017, 2020).

Damaged mitochondria are removed by a selective autophagic process, so-called mitophagy. Compromised mitophagy by genetic knockout of *Parkin, PINK1*, or *Irgm1* accumulates cytosolic mtDNA and subsequently leads to inflammatory and IFN responses by cGAS‒STING signaling, which promotes the progression of neurodegeneration or systemic autoimmunity, respectively (Sliter et al., 2018; Jena et al., 2020; Rai et al., 2021). Overall, these studies suggest that cGAS is the primary sensor of mtDNA stress and subsequent signaling promotes the progression of multiple sterile inflammatory diseases. In-depth research is required to determine whether targeting this pathway represents an effective therapeutic strategy for these diseases.

### Genomic DNA

Although the vast majority of cGAS locates in the nucleus, it is usually inaccessible to self-genomic DNA, as the nuclear cGAS is tethered tightly by histone H2A and H2B in the nucleosomes (Jiang et al., 2019; Volkman et al., 2019). High-affinity nucleosome binding blocks genomic DNA binding and prevents cGAS-induced autoimmunity. Accordingly, mutation of these key residues in the cGAS–histone binding site abrogates nucleosome binding and promotes nuclear DNA-dependent cGAS activation (Boyer et al., 2020; Cao et al., 2020; Kujirai et al., 2020; Michalski et al., 2020; Pathare et al., 2020; Zhao et al., 2020a). Misprocessing of canonical histone transcripts in a subset of Aicardi–Goutières syndrome (AGS) patients with biallelic mutations in *LSM11* and *RNU7-1* specifically disturbs linker histone stoichiometries and leads to type I IFN signatures by cGAS redistribution and activation within the nucleus (Uggenti et al., 2020). Similarly, barrier-to-autointegration factor 1 (BAF1) also restricts cGAS activity by dynamically outcompeting cGAS for nuclear DNA binding. Suppression of *BAF1* increases the accumulation of cGAS within discrete intranuclear foci and induces a robust IFN response, which may be involved in the pathogenesis of autoimmune diseases (Guey et al., 2020).

Apart from DNA sensing in the nuclear, genomic DNA leaks to the cytosol and becomes accessible to cytosolic cGAS in the context of DNA damage, cellular senescence, or derepression of retroelements that promotes disease progression. DNA damage caused by 7,12-dimethylbenz(*a*)anthracene, cisplatin, and etoposide induces nuclear DNA leakage into the cytosol, which activates STING-dependent inflammation and drives skin carcinogenesis (Ahn et al., 2014). Inefficient DNA damage repair caused by ataxia‒telangiectasia (A–T) mutated dysfunction leads to the accumulation of cytoplasmic fragments of genomic DNA, which activates STING signaling in microglia and contributes to A‒T neurodegeneration (Song et al., 2019). Cellular senescence is a stress response characterized by the permanent growth arrest of damaged or aged cells. Loss of nuclear lamina protein B1 in senescent cells disrupts the integrity of the nuclear envelope, which causes the accumulation of chromatin fragments into the cytoplasm (micronuclei). Cytosolic chromatin fragments drive the production of the senescence-associated secretory phenotype by activation of the cGAS‒STING pathway, which is critical for senescence-associated effects on ageing and age-related disorders (Dou et al., 2017; Gluck et al., 2017). Endogenous retroelements are a special class of genetic elements that can integrate into the human genome. Long-interspersed element-1 (LINE1) is the only known human retrotransposable element capable of autonomous retrotransposition. While generally silenced, LINE1 activity becomes transcriptionally derepressed during aging and, subsequently, the accumulation of cytosolic LINE1 cDNA initiates age-associated inflammation by activation of the cGAS‒STING pathway (De Cecco et al., 2019; Simon et al., 2019). Additionally, TREX1 (a cytosolic 3′→5′ exonuclease), RNase H2 complex (a ribonuclease complex), and SAMHD1 (a dNTP triphosphohydrolase) are known LINE1 repressors and, when mutated in AGS, cause type I IFN signatures and inflammatory injuries through cGAS‒STING signaling (Crow et al., 2015; Gao et al., 2015; Pokatayev et al., 2016; Coquel et al., 2018). Taken together, DNA damage and/or gene mutations in DNA/cGAS restriction factors under various disease conditions activate cGAS signaling in both the cytoplasm and nucleus upon DNA binding, which induces cGAS-dependent autoimmunity and exacerbates cGAS-associated sterile inflammation. Consequently, it is of great significance to explore cGAS-specific antagonists and evaluating their efficacies in these diseases may lead to the discovery of novel therapeutics.

## Ligand-independent STING signaling in sterile inflammatory diseases

**Figure 2:**
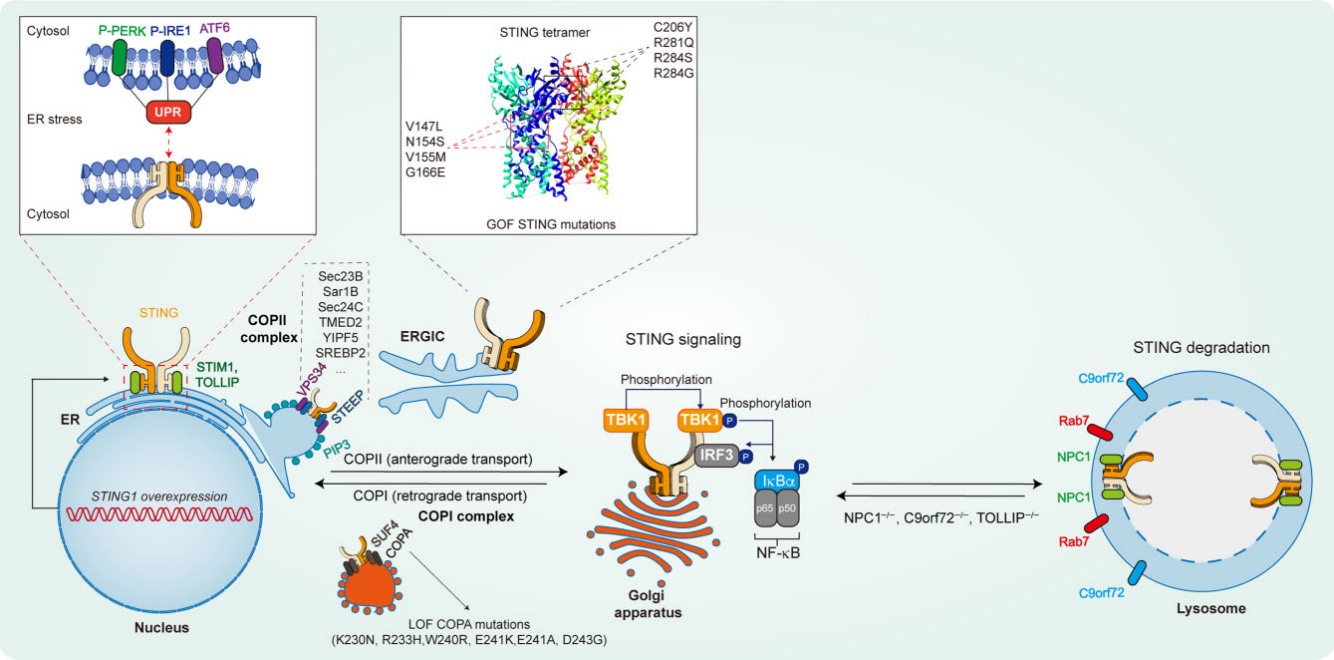
Ligand-independent STING signaling in sterile inflammatory diseases. STING localizes in the ER in the resting state and is stabilized by ER-retention factors (STIM1 and TOLLIP). STING trafficking from the ER to the Golgi is mediated by COPII and COPI complexes. After executing the cellular functions, STING exits the Golgi and eventually transports into the lysosome for degradation. Disrupting STING cofactors in the ER or the lysosome, ER stress, GOF STING mutations, and LOF COPA mutations all lead to sustained STING signaling, which promotes the progression of multiple sterile inflammatory diseases.

STING presents as a unique dimeric structure in the resting state, which generates a V-shaped ligand-binding pocket for CDN recognition by the cytoplasmic domains (138‒379 amino acids) (Huang et al., 2012; Ouyang et al., 2012; Shang et al., 2012; Shu et al., 2012; Yin et al., 2012). 2′3′-cGAMP binding induces a closed conformation of the STING dimer and leads to a 180° clockwise rotation of its cytoplasmic domains relative to the transmembrane domain (1‒137 amino acids) (Shang et al., 2019). This conformational switch of the STING dimer generates a surface geometry that promotes the STING dimer‒dimer interaction and subsequent formation of STING oligomers in a side-by-side configuration (Ergun et al., 2019; Shang et al., 2019). STING oligomers exit the ER toward the ER–Golgi intermediate compartment (ERGIC) and the Golgi, where the signaling transduces to IRF3 activation for immunoreactions, NF-κB activation for inflammatory responses, and LC3 lipidation for autophagy induction (Hopfner and Hornung, 2020). After executing these cellular functions, STING exits the Golgi and eventually transports into the lysosome for degradation (Gonugunta et al., 2017). The mechanism of this ligand-dependent STING activation is well characterized, whereas the understanding of ligand-independent STING activation is still in the preliminary phase and increasing studies prove that the disturbance of STING oligomerization, trafficking, and stability leads to its autoactivation in multiple sterile inflammatory diseases (Figure 2). In this section, we review the recent progress of this active area and discuss how ligand-independent STING signaling is involved in disease progression.

### Gain-of-function STING1 mutations

The earliest discovered cases of ligand-independent STING activation are gain-of-function (GOF) *STING1* (also known as *TMEM173*) mutations in a rare autoinflammatory disease named STING-associated vasculopathy with onset in infancy (SAVI) (Jeremiah et al., 2014; Liu et al., 2014). Subsequently, several mutated residues are identified in two separate regions on STING, namely the dimerization interface (V147L, N154S, V155M, and G166E) and the polymerization interface (C206Y, R281Q, R284S, and R284G) (Konig et al., 2017; Melki et al., 2017; Konno et al., 2018). Structural analysis suggests that V147L, N154S, and V155M substitutions induce a 180° rotation of the cytoplasmic domains, G166E substitution enhances STING dimer stabilization, and C206Y, R281Q, R284S, and R284G mutants relieve the autoinhibition of STING oligomerization, thus causing spontaneous STING oligomerization and accumulation within the Golgi apparatus without 2′3′-cGAMP binding (Ergun et al., 2019; Shang et al., 2019). The autoactivation of these STING mutants in SAVI patients leads to severe lung inflammation, widespread skin vasculopathy, systemic inflammation, combined immunodeficiency, and premature death (Fremond et al., 2021). Reconstructing these clinical manifestations in STING N153S and V154M knock-in mice reveals the importance of IFN-independent activities of STING activation in controlling lung inflammation, myeloid cell expansion, and lymphopenia (Warner et al., 2017; Motwani et al., 2019a). Further transcriptomic analysis confirms widespread IFN-independent STING activities in macrophages and T cells that associate with antigen presentation and cell death, respectively (Wu et al., 2020). Notably, ablation of type I IFN signaling fails to rescue the lethality in V154M mice (Motwani et al., 2019a). Consistently, a recent clinical study of SAVI patients with V155M mutation in China reports that one patient died after treatment with tofacitinib (JAK1/3 inhibitor) (Tang et al., 2020). Thus, STING-specific antagonists that block both IFN-dependent and IFN-independent activities may be superior to JAK inhibitors in the treatment of SAVI patients. The discovery of novel STING antagonists with high efficacy, safety, and suitability for clinical application is urgently needed.

### Trafficking-mediated STING activation

The trafficking of STING from the ER to the ERGIC and the Golgi is essential for its function. In the resting state, STING localizes to the ER membrane through its transmembrane domain and holds the position by interacting with ER-retention factors to maintain immunological quiescence. Stromal interaction molecule 1 (STIM1) acts as an ER-retention factor that anchors STING on the ER and limits STING signaling (Srikanth et al., 2019). Both LOF mutation in STIM1 (E136X) and GOF mutations in STING (V147L, N154S, and V155M) disrupt STIM1‒STING interaction and lead to STING localization at the ERGIC, which induces autoimmune complications in patients (Srikanth et al., 2019). Following STING activation, trafficking cofactors in the STING complex promote its translocation from the ER to other organelles. More specifically, the STING ER exit protein (STEEP) recruits the phosphatidylinositol 3 (PI3) kinase‒VPS34 complex I to produce PI3-phosphate (PI3P) within the ER membrane. Accumulation of PI3P induces the formation of membrane curvature in the STING-containing areas to recruit the coatomer protein complex II (COPII) that facilitates STING trafficking from the ER to the Golgi (Gui et al., 2019; Zhang et al., 2020a). Elevated STEEP interaction with SAVI-associated STING variants participates in ligand-independent STING activation and type I IFN signatures in SAVI patients (Zhang et al., 2020a). Likewise, transmembrane emp24 protein transport domain containing 2 and YIP1 family member 5 also facilitate COPII-dependent STING trafficking and their function in STING-associated autoimmune diseases needs further investigation (Sun et al., 2018; Ran et al., 2019). Additionally, sterol regulatory element-binding protein 2 (SREBP2) activation induced by cholesterol deficiency in the ER primes STING signaling through ‘tethering’ the trafficking of STING, which is required for neuropathology in Niemann–Pick disease type C (Chu et al., 2021b). Intriguingly, STING may continuously cycle between the ER and the Golgi in the resting state by COPI-mediated retrograde transport. Missense mutations in *COPA* impair STING retrieval and force it to accumulate and activate at the Golgi, which is critical for the pathogenesis of COPA syndrome (Deng et al., 2020; Lepelley et al., 2020; Mukai et al., 2021). The clinical and histopathological similarities between COPA syndrome and SAVI further highlight the importance of ligand-independent STING activation in the development of multiple autoimmune diseases.

### Abnormal STING stability

As prolonged STING signaling induces an excessive immune and inflammatory response that may be harmful to the host, STING is tightly regulated to ensure timely response against infection and quickly degraded after activation to avoid tissue injury. STING stability is largely controlled by trafficking-mediated lysosomal degradation and ubiquitin-mediated proteasomal degradation (Hopfner and Hornung, 2020). Several E3 ubiquitin ligases, including ring finger protein 5, tripartite motif protein 29 (TRIM29), and TRIM30α, are induced during viral infection, which interact with STING and catalyze its Lys48-linked polyubiquitination for degradation in a proteasome-dependent pathway (Zhong et al., 2009; Wang et al., 2015; Xing et al., 2017). Although proteasome-dependent STING degradation is crucial for host cells to control excessive innate immune response after viral infection, it seems to be dispensable in STING-associated sterile inflammatory diseases. In contrast, preventing lysosome-dependent STING degradation leads to ligand-independent STING activation and promotes the progression of several sterile inflammatory diseases. In the resting state, Toll-interacting protein (TOLLIP) interacts with STING and stabilizes STING protein on the ER by competing with the serine/threonine protein kinase/endoribonuclease inositol-requiring enzyme 1α (IRE1α)‒lysosome-mediated STING degradation (Pokatayev et al., 2020). At the late stage of its activation, STING traffics to the lysosome for degradation through autophagosomes and endosomes in a RAB7 GTPase-dependent manner (Gonugunta et al., 2017; Gui et al., 2019). Accordingly, blocking STING degradation by lysosome inhibitors (bafilomycin A1 or chloroquine) enhances STING activation and downstream immune gene expression (Gonugunta et al., 2017; Gui et al., 2019). Following a recent study with spatiotemporally resolved proximity labelling and quantitative proteomics, a number of STING interactors enriched in the ER, ERGIC/Golgi, endosome, and lysosome during STING activation have been identified (Chu et al., 2021b). Further investigating into a lysosomal candidate protein Niemann–Pick type C1 (NPC1) demonstrates its crucial role in both STING degradation by recruiting STING to the lysosome and STING trafficking by blocking SREBP2‒STING tethering (Chu et al., 2021b). Notably, pathological features of neuroinflammation, neuropathology, and motor defects in human Niemann–Pick disease type C reconstructing in *Npc1*^−/−^ mice are significantly improved by genetic deletion of *Sting* or *Irf3*, but not deletion of *Cgas*, which indicates the predominant function of ligand-independent STING activation in disease progression and that pharmacological inhibition of STING activation maybe a promising strategy for the treatment of Niemann–Pick disease type C (Chu et al., 2021b). Furthermore, disrupting lysosomal degradation of STING in *C9orf72*^−/−^ myeloid cells increases STING activity and drives chronical elevation of the type I IFN signature, which induces systemic autoinflammation in *C9orf72*^−/−^ mice (McCauley et al., 2020). Consistently, expansion of a hexanucleotide repeat (GGGGCC) in the *C9orf72* gene of patients with familial ALS and frontotemporal dementia decreases C9orf72 protein expression and induces type I IFN response in blood-derived macrophages, whole blood, and brain tissue in a STING-dependent manner (McCauley et al., 2020).

From the beginning of discovery, increased STING expression and activation have been noted by overexpression of STING in cells that induces robust expression of IFNs and inflammatory cytokines (Ishikawa and Barber, 2008; Zhong et al., 2008; Sun et al., 2009). Correspondingly, elevated STING expression and activity in nonalcoholic fatty liver disease, acute pancreatitis, ischemic stroke, osteoarthritis, intervertebral disc degeneration, and traumatic brain injury exacerbate the progression of these diseases (Luo et al., 2018; Zhao et al., 2018; Li et al., 2020; Sen et al., 2020; Guo et al., 2021a, b). Specifically, overexpression of STING induces NF-κB activation in chondrocytes, which promotes degradation of the extracellular matrix, apoptosis, and senescence (Guo et al., 2021a, b). This type of STING activation may result from the increased oligomerization and translocation induced by elevated STING expression.

### ER stress

ER protein homeostasis is maintained by unfolded protein response (UPR), a network of signal transduction pathways to reprogram gene transcription, mRNA translation, and protein modifications that ensure protein folding fidelity and maintain ER functions (Hetz et al., 2020). Abnormal accumulation of unfolded or misfolded proteins in the ER lumen leads to ER stress. Three major UPR branches mediated by the ER transmembrane protein IRE1α, protein kinase R-like endoplasmic reticulum kinase (PERK), and activating transcription factor 6 are activated under ER stress, which sense the protein folding status in the ER lumen and transmit this information across the ER membrane to the cytosol (Hetz et al., 2020). If ER stress fails to resolve, sustained UPR engages a distinct set of proapoptotic programs that contribute to cell death. Increasing studies suggest that ER stress promotes STING activation, and vice versa (Smith, 2020). The reciprocal cross-regulation between ER stress and STING accelerates the progression of several sterile inflammatory diseases. In mouse models of alcoholic liver disease and liver fibrosis, acute/short-term alcohol and CCl_4_ administration-induced ER stress activates proapoptotic signal transduction through the STING‒TBK1‒pIRF3‒Bax cascade, which contributes to hepatocyte apoptosis (Petrasek et al., 2013; Iracheta-Vellve et al., 2016). During traumatic brain injury, neuronal ER stress initiated by PERK phosphorylation activates STING‒IRF3 signaling and subsequently IFN-β expression that promotes microglia activation and T cell infiltration, which eventually lead to neuroinflammation and white matter injury (Sen et al., 2020). The reciprocal crosstalk between ER stress and STING has also been revealed in pathological cardiac hypertrophy, where aortic banding-induced ER stress, as evidenced by elevated expression of p-PERK and p-eIF2α, is markedly attenuated by STING deletion. Furthermore, angiotensin II-induced STING expression and activation could be augmented by ER stress activators, whereas they were markedly abolished by ER stress inhibitors (Zhang et al., 2020b). Through structure and function analysis between STING and ER stress, a UPR motif within STING (322–343 amino acids), specifically residues Arg331 and Arg334, was defined to be essential for STING-mediated UPR. Thus, STING activation augments ER stress induced by T-cell receptor signaling in T cells and primes cell death (Wu et al., 2019). Nevertheless, the detailed molecular basis for the reciprocal activation between STING and ER stress is largely unknown and remains to be fully elucidated.

## Discovery of cGAS and STING antagonists

**Figure 3:**
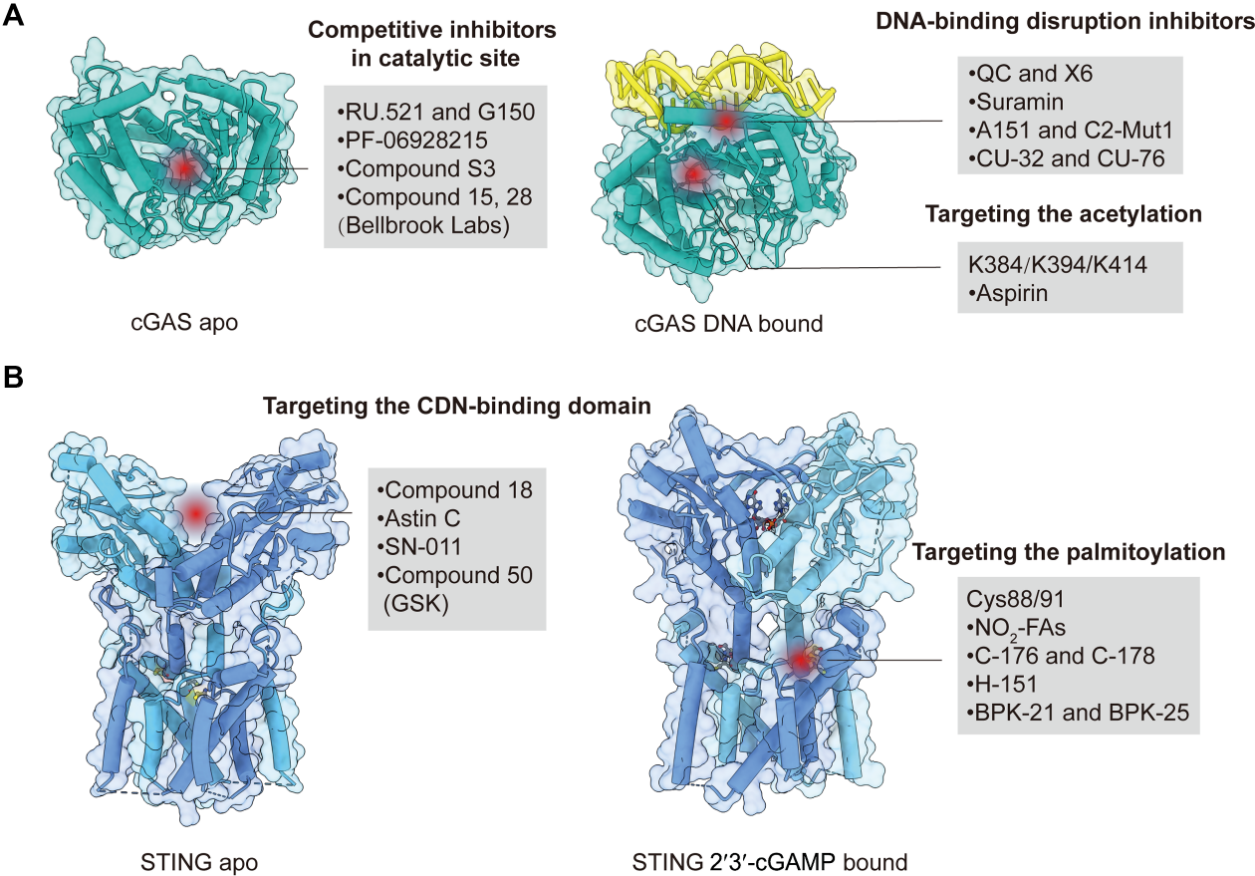
Mechanisms of cGAS and STING inhibitors. (**A**) Inhibitors competing with enzyme substrates for binding to the cGAS catalytic pocket are shown on the left (PDB: 4O68), and those reported to disrupt the interaction between DNA and cGAS or induce cGAS acetylation are shown on the right (PDB: 6CT9). (**B**) Inhibitors targeting the CBD of STING in the apo state are shown on the left (PDB: 6NT5). By recognition of 2′3′-cGAMP, STING undergoes conformational rearrangements (PDB: 6NT7), and inhibitors impeding palmitoylation in Cys88/91 of STING are shown on the right. PDB, protein data bank.

As mentioned above, both cGAS-dependent and cGAS-independent STING signaling can be involved in the progression of multiple sterile inflammatory diseases. Therefore, inhibiting cGAS and STING activation has promising therapeutic potential, and a variety of strategies have been adopted to develop novel cGAS and STING antagonists. Here, we discuss the structure-based drug discovery on cGAS and STING. The chemical structures and preclinical efficacies of specific antagonists targeting cGAS (Table 1) and STING (Table 2) are summarized and the inhibitory mechanisms of these agents are further illustrated (Figure 3).

**Table 1:** Compound structures of described cGAS inhibitors.

Compound	Structure	Inhibitory potency	Tested species	Reference
QC	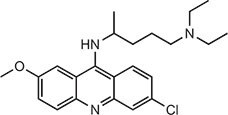	IC_50_ = 13 μM, cGAS enzymatic assay; IC_50_ = 3.7 μM, THP-1 cells	Human	An et al. (2015)
X6	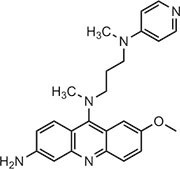	IC_50_ = 14 μM, THP-1 cells	Human/mouse	An et al. (2018)
Suramin	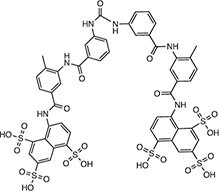	NA	Human	Wang et al. (2018)
A151	5′-TTAGGGTTAGGGTTAGGGTTAGGG-3′	IC_50_ = 165 nM, THP-1 cells	Human/mouse	Steinhagen et al. (2018)
C2-Mut1	5′-mG*mC*mG*mG*mU*A*T*C*C*A*T*G*T*C*C*mC*mA*mG*mG*mC-3′	IC_50_ = 56 nM, THP-1 cells	Human/mouse	Valentin et al. (2021)
CU-32	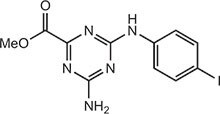	IC_50_ = 0.45 μM, cGAS enzymatic assay	Human/mouse	Padilla-Salinas et al. (2020)
CU-76	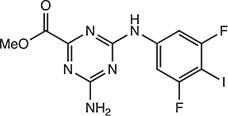	IC_50_ = 0.24 μM, cGAS enzymatic assay	Human/mouse	Padilla-Salinas et al. (2020)
RU.521	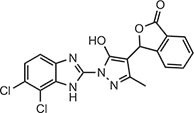	IC_50_ = 700 nM, RAW cells	Human/mouse	Vincent et al. (2017)
G150	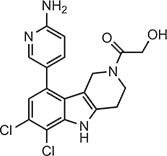	IC_50_ = 10.2 nM, cGAS enzymatic assay; IC_50_ = 1.96 µM, THP-1 cells	Mouse	Lama et al. (2019)
PF-06928215	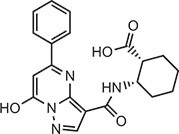	IC_50_ = 4.9 μM, cGAS enzymatic assay	Human	Hall et al. (2017)
Compound S3	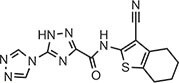	IC_50_ = 4.9 ± 0.26 μM, peptidyl-prolyl cis/trans isomerase-coupled assay	Human	Zhao et al. (2020b)
Aspirin	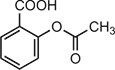	IC_50_ = 0.760 mM, BMDMs from *Trex1*^–/–^ mice	Human/mouse	Dai et al. (2019)
Perillaldehyde	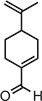	IC_50_ = 31.3 µM, cGAS enzymatic assay; IC_50_ = 55.79 ± 3.25 µM, HFF cells	Human/mouse	Chu et al. (2021a)
Compound 15 (Bellbrook Labs)	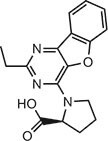	IC_50_ = 0.356 µM, cGAS enzymatic assay	Human	Lowery et al. (2020a)
Compound 28 (Bellbrook Labs)	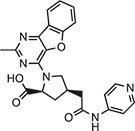	IC_50_ = 13 µM, THP-1 cells	Human	Lowery et al. (2020a)
Compound 14 (Bellbrook Labs)	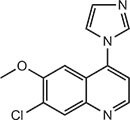	IC_50_ = 0.391 µM, cGAS enzymatic assay; IC_50_ = 68 µM, THP-1 cells	Human	Lowery et al. (2020b)
C1089 (Aduro Biotech)	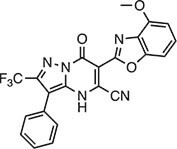	IC_50_ < 20 µM, THP-1 cells	Human	Ndubaku et al. (2019)
TA1065 (Aduro Biotech)	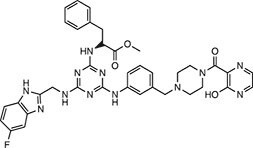	IC_50_ = 20‒100 µM, THP-1 cells	Human	Katibah et al. (2019)
Compound 6 (Aduro Biotech)	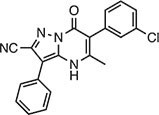	IC_50_ < 1 µM, cGAS enzymatic assay; IC_50_ < 10 µM, THP-1 cells	Human	Ciblat et al. (2020)

‘m’ indicates 2′OMe base and ‘*’ denotes the phosphorothioate backbone. NA, not available. IC_50_, the half maximal inhibitory concentration.

**Table 2:** Compound structures of described STING inhibitors.

Compound	Structure	Inhibitory potency	Test species	Reference
Compound 18	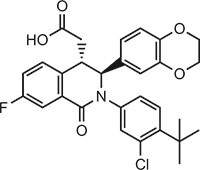	IC_50_ = 11 μM, THP-1 cells	Human	Siu et al. (2019)
Astin C	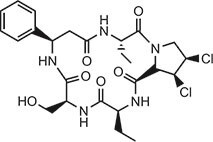	Isothermal titration calorimetry *K*_d_ = 53 nM	Human/mouse	Li et al. (2018)
SN-011	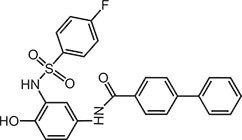	IC_50_ = 76 nM, L929 cells; IC_50_ = 502.8 nM, HFFs	Human/mouse	Hong et al. (2021b)
Nitro-fatty acids	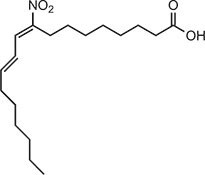	NA	Human/mouse	Hansen et al. (2018)
C-176	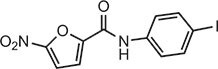	NA	Mouse	Haag et al. (2018b)
C-178	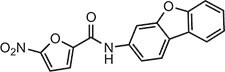	NA	Mouse	Haag et al. (2018b)
H-151	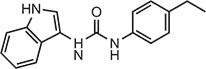	IC_50_ ∼ 100 nM, MEFs and HFFs	Human/mouse	Haag et al. (2018b)
BPK-21	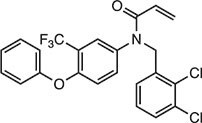	NA	Human	Vinogradova et al. (2020)
BPK-25	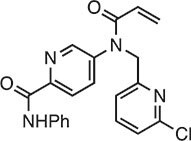	IC_50_ = 3.2 μM, THP-1 cells	Human	Vinogradova et al. (2020)
Compound 50 (GSK)	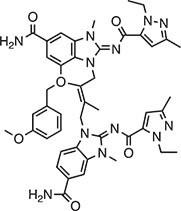	pIC_50_ = 8.9, THP-1 cells; pIC_50_ = 7.1, hPBMCs	Human	Fosbenner et al. (2019)
Compound 275 (IFM)	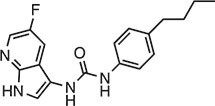	IC_50_ = 0.04‒0.2 µM, THP-1 cells	Human	Roush et al. (2020c)
Compound 147 (IFM)	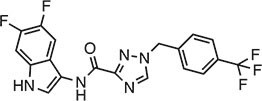	IC_50_ = 0.04‒0.2 µM, THP-1 cells	Human	Seidel et al. (2020)
Compound 118 (IFM)	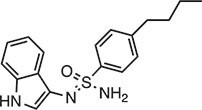	IC_50_ = 0.04‒0.2 µM, THP-1 cells	Human	Venkatraman et al. (2020c)
Compound 208 (IFM)	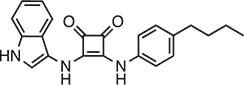	IC_50_ = 0.2‒1.0 µM, THP-1 cells	Human	Venkatraman et al. (2020b)

NA, not available. IC_50_, the half maximal inhibitory concentration.

### Inhibitors targeting cGAS

The C-terminal domain of cGAS (161‒522 amino acids) acts as the nucleotidyltransferase domain and contains positively charged DNA-binding sites. The sugar–phosphate backbone of DNA binds to the primary site (site A and site B) of cGAS, which induces a global conformational change (Civril et al., 2013). The rearranged catalytic pocket of cGAS then forms an optimal structure for the recognition of ATP and GTP substrates and production of 2′3′-cGAMP (Gao et al., 2013a). As such, inhibitors targeting cGAS protein are identified to (i) disrupt the interaction between DNA and cGAS or compete with DNA binding as DNA analogs, (ii) occupy the cGAS catalytic pocket to block the binding of substrates and the product synthesis, and (iii) mediate PTMs of cGAS.

#### DNA-binding disruption inhibitors

Hydroxychloroquine (HCQ) and quinacrine (QC) are the first reported cGAS inhibitors disturbing cGAS‒DNA interaction (An et al., 2015). The *in silico* docking predicted that HCQ interacts with the residues Lys372 and Arg342, which mediates the stability of the cGAS/DNA complex. However, HCQ and QC impair cell viability at high doses and inhibit RIG-I signaling activation and cellular pyroptosis. Due to poor safety and target specificity, the second-generation antimalarial-like drug X6 was developed, which included novel aminoacridine derivatives with positively charged amine groups (An et al., 2018). X6 was predicted to locate in the minor groove at the cGAS/DNA interface. Importantly, treatment with X6, but not HCQ, significantly reduces IFN-β and ISG expression in *Trex1*^–/–^ mice and PBMCs from SLE patients. Because of the absence of crystal structure and detailed pharmacological data, the therapeutic potential of these compounds needs further research. Suramin, initially used for the treatment of river blindness and African sleeping sickness, was recently identified to inhibit cGAS activity (Wang et al., 2018). While suramin competes with dsDNA for cGAS binding, it also suppresses the IL-6 expression in THP-1 cells stimulated by polyinosine–polycytidylic acid (poly(I:C)), which contributes to the unexpected off-target effects. Additionally, by performing a high-throughput virtual screening and structure‒activity relation analysis of hit compounds, CU-32 and CU-76 were identified as novel cGAS antagonists (Padilla-Salinas et al., 2020). Molecular docking study of CU-76 indicates that it might bind in the groove at the interface of cGAS dimer, which disturbs the cGAS dimerization. The exact inhibitory mechanism of these compounds needs to be further clarified.

Beyond these small-molecule inhibitors, some oligonucleotides also compete with DNA for binding to cGAS. Synthetic oligonucleotides (ODNs) containing repetitive TTAGGG motifs (ODN A151) were reported to block cGAS-mediated type I IFN response induced by dsDNA and *Trex1* deficiency (Steinhagen et al., 2018). In addition, the chemical-optimized oligonucleotides, e.g. C2-Mut1, potently inhibit cGAS activation, presumably as inactive competitors of DNA binding in a sequence-dependent manner (Valentin et al., 2021). However, these suppressive oligonucleotides likely have similar off-target problems, for the universal protein‒DNA binding mechanism within cells.

#### Competitive inhibitors in the catalytic site

Through a RapidFire mass spectrometry system and high-throughput screen, RU.521 was identified as the first competitive inhibitor targeting the cGAS catalytic site (Vincent et al., 2017). The co-crystal structure proved that two modified chlorines in RU.521 target deeper in the cGAS catalytic pocket, which increases the stacking surface of RU.521 with Arg364 and Tyr421. Despite a strong potency in a mouse cell line, RU.521 is a poor inhibitor of human cGAS (h-cGAS). Compound G150 was further identified as a specific h-cGAS inhibitor through a high-throughput screen using the h-cGAS-based chemiluminescence assay (Lama et al., 2019). Notably, it is still difficult to rationalize the inhibitor optimization pathway due to the limited steric constraints and factoring in water and metal ion networks by partially occupying G150 in the cGAS binding pocket.

PF-06928215 is another competitive cGAS inhibitor in the catalytic site, which was identified through a saturation transfer difference-based screening against the Pfizer fragment library (Hall et al., 2017). The structure of PF-06928215 with h-cGAS indicates that this compound might mimic the interaction by which cGAS holds the linear intermediate of 2′3′-cGAMP during the catalytic process in the active site. Despite the great potency in biochemical assays, PF06928215 and its derivatives lost the inhibitory activity in cellular assays. It was proposed that the high concentration of ATP and GTP within the cytoplasm might contribute to this low potency *in vitro*. Subsequently, a virtual screening model was built based on the crystal structure of h-cGAS in complex with PF06928215 (Zhao et al., 2020b). Compounds S2 and S3 were identified as cGAS inhibitors through the virtual screening (Zhao et al., 2020b). The biological activities of these compounds in cells and disease models need further evaluation.

#### Inhibitors targeting the acetylation site

Acetylation of cGAS on Lys384, Lys394, and Lys414 has been identified in quiescent cells and deacetylation of cGAS by histone deacetylase 3 upon DNA treatment is involved in cGAS activation (Dai et al., 2019). Interestingly, aspirin, a nonsteroidal anti-inflammatory drug, was identified to modify the inhibitory acetylation on cGAS at Lys384, Lys394, and Lys414. Consistently, aspirin is effective in suppressing DNA-mediated autoimmunity in both *Trex1*^–/–^ mice and PBMCs from AGS patients.

#### Inhibitors disclosed by patents

A series of tricyclic benzofuropyrimidine analogs were identified as cGAS inhibitors disclosed by a Bellbrook Labs patent (Lowery et al., 2020a). Structural analysis revealed that Tyr436 and Arg376 in human cGAS are essential for Compound 15 binding. Further structural optimization leads to the discovery of Compound 28 with improved inhibitory potency. Another patent disclosed by Bellbrook Labs identified Compound 14 as a cGAS antagonist with unknown mechanism (Lowery et al., 2020b). Aduro Biotech also disclosed various compounds with inhibitory activity on cGAS, among which TA1065 has a triazine core, C1089 consists of a pyrazolopyrimidinone core, and Compound 6 possesses an imidazopyridazinone core (Katibah et al., 2019; Ndubaku et al., 2019; Ciblat et al., 2020).

### Inhibitors targeting STING

As mentioned above, either ligand-dependent or ligand-independent STING activation promotes the progression of sterile inflammatory diseases. Several small-molecule compounds have been identified to occupy the STING CDN-binding pocket for inhibiting ligand-dependent STING activation. Besides, interrupting STING oligomerization, translocation, and signal transduction is effective in blocking STING activation with or without ligand binding. The STING polymer interface stabilized by salt bridge interaction between Asp301 and Arg281 or Arg284 is an attractive pocket for compound binding and drug screening. Disrupting the interaction between STING and its trafficking cofactors by specific chemical molecules also represents a reasonable strategy for the discovery of STING antagonists. Additionally, STING PTMs, including palmitoylation, ubiquitination, and phosphorylation, are essential for recruiting and activating TBK1 and IRF3, and thus pharmacologically blocking the progress of these critical PTMs may cut off downstream signal transduction.

#### Inhibitors in the CDN-binding pocket

Compound 18 is the first confirmed STING inhibitor targeting the CDN-binding domain (CBD) (Siu et al., 2019). Crystallographic study of Compound 18 with STING revealed that two molecules of the compound bind to a single STING homodimer in the inactive ‘open’ conformation. While Compound 18 exhibits good oral exposure, it is a weak STING antagonist when tested *in vitro*. Astin C is another reported STING antagonist in the CDN-binding pocket, as revealed by docking analysis (Li et al., 2018). Interestingly, astin C specifically blocks the recruitment of IRF3 onto the STING signalosome while keeping the STING‒TBK1 interaction intact. Recently, an *in silico* docking screen against a STING CDN-binding pocket led to the discovery of SN-011 as a novel STING antagonist with promising therapeutic potential (Hong et al., 2021b). SN-011 significantly reduces IFN and inflammatory cytokine induction activated by 2′3′-cGAMP, *Trex1* deficiency, or SAVI-associated STING mutants. Consistently, SN-011 strongly inhibits systemic inflammation and protects *Trex1*^−/−^ mice from death.

#### Inhibitors targeting the palmitoylation site

Palmitoylation of STING (Cys88/91) in the Golgi apparatus by palmitoyltransferases is essential for signaling transduction (Mukai et al., 2016). Accumulated endogenous nitro-fatty acids (NO_2_-FAs) during virus infection covalently modify STING by nitroalkylation at its palmitoylation site, thus preventing STING activation (Hansen et al., 2018). Moreover, a series of nitrofuran derivatives, including C-176, C-178, and H-151, have been identified through a cell-based chemical screen, which covalently target STING at Cys91 and block the activation-induced palmitoylation of STING (Haag et al., 2018). These compounds have been widely evaluated in disease models and proved to be effective in ameliorating multiorgan inflammation in AGS, ALS, Niemann–Pick disease type C, chronic kidney disease, and psoriasis (Haag et al., 2018; Chung et al., 2019; Yu et al., 2020; Chu et al., 2021b; Pan et al., 2021). Notably, C-176 and C-178 are specific mouse STING inhibitors, while H-151 inhibits both mouse and human STING activation. Importantly, >3000 covalently liganded cysteines have been characterized on functionally and structurally diverse proteins in human T cells by fishing with electrophilic small-molecule fragments and subsequently chemical proteomic analysis (Vinogradova et al., 2020). The compounds BPK-21 and BPK-25 were screened out to suppress T cell activation through distinct mechanisms, including interacting with Cys91 in STING, Cys203 in MYD88, and/or Cys342 in excision repair cross-complementation group 3. These findings underscore the potential off-target risks of these STING palmitoylation inhibitors and it is particularly important to assess drug safety in the development of these compounds.

#### Inhibitors disclosed by patents

GlaxoSmithKline released a series of patents protecting STING antagonists, which were derived from previously reported amidobenzimidazole (ABZI) STING agonists that compete with 2′3′-cGAMP for binding to the CBD of STING (Ramanjulu et al., 2018; Fosbenner et al., 2019). Similarly to diABZIs, introducing a linker between two molecules in Compound 50 dramatically enhanced its inhibitory activity by competing with 2′3′-cGAMP binding. IFM therapeutics disclosed a series of STING antagonists in recent patent applications (Katz et al., 2020; Roush et al., 2020a, b, c; Seidel et al., 2020; Venkatraman et al., 2020a, b, c). Structural analysis indicated that these highly potent compounds (Compound 275, Compound 147, Compound 118, and Compound 208 in Table 2) in the newly disclosed patents are derived from H-151 with the optimization of chemical structures.

## Conclusions and future perspectives

Significant progress has been made in dissecting the mechanisms that lead to the overaction of the cGAS‒STING pathway under pathological conditions and to what degree it participates in disease progression. Although a wide variety of sterile inflammatory diseases have been summarized in this review, it should be noted that evaluating these specific cGAS and STING antagonists in the diseases caused by primary cGAS‒STING activation is more reasonable and urgent than in the diseases aggravated by secondary cGAS‒STING activation, especially at this preliminary stage of drug discovery. Additionally, as the core regulatory protein, STING, not only accepts the damaged signal from host-derived DNAs but also responds to gene mutations and ER stress, which ultimately leads to sterile inflammation and tissue injury involved in the development of multiple autoimmune and inflammatory diseases. From this point, targeting STING may represent a preferable strategy over targeting cGAS in drug discovery. Based on the structure of the parent nucleus of these identified lead compounds, chemical optimization is necessarily needed to improve their bioactivity, target selectivity, and pharmaceutical absorption, while minimizing toxic effects. Moreover, from the perspective of bioactivity, discovered antagonists or inhibitors from biochemical-based high-throughput screening might be compounds without effect on cell-based assays. Therefore, drug screening with a combined cell-based high-throughput method would be preferred in the future. Promisingly, new biotechnologies, such as proteolysis-targeting chimeras leading to protein degradation and RNA-targeting small molecules, might provide alternative approaches to block cGAS‒STING signaling and offer more clinical choices for these sterile inflammatory diseases in which cGAS‒STING signaling is implicated.

## Notes

**Author contributions:** Z.H. and J.M. drafted the manuscript text and prepared figures. H.G., J.Z., and C.W. edited the paper and reviewed its final version.
